# Changes in Inflammatory Cytokines in Saliva after Non-Surgical Periodontal Therapy: A Systematic Review and Meta-Analysis

**DOI:** 10.3390/ijerph18010194

**Published:** 2020-12-29

**Authors:** Ji-Youn Kim, Han-Na Kim

**Affiliations:** 1 Department of Dental Hygiene, College of Health Science, Gachon University, Incheon 21936, Korea; hoho6434@gachon.ac.kr; 2 Department of Dental Hygiene, College of Health and Medical Sciences, Cheongju University, Cheongju 28503, Korea

**Keywords:** periodontitis, scaling, saliva, cytokine, biomarker

## Abstract

To determine the diagnostic value of inflammatory cytokines in periodontal disease, we performed a systematic review of the changes in inflammatory cytokines after non-surgical periodontal therapy and a meta-analysis of the utility of interleukin (IL)-1β and matrix metalloproteinase (MMP)-8 as salivary biomarkers. All available papers published in English until 20 August 2020, were searched in the MEDLINE and EMBASE databases. Population, intervention, comparison, and outcome data were extracted from the selected studies, and the roles of IL-1β and MMP-8 were assessed in a meta-analysis. Eleven studies, including two meta-analyses, were assessed in the systematic review. Biomarkers showing high levels in periodontal disease were salivary IL-1β, IL-4, IL-6, MMP-8, and tissue inhibitor of matrix metalloproteinases (TIMP)-2, and those in the controls were tumor necrosis factor (TNF)-α, IL-10, IL-17, and IL-32. Biomarkers that decreased after scaling and root planning (SRP) and oral hygiene instruction (OHI) in periodontitis patients were IL-1β, MMP-8, MMP-9, prostaglandin E2 (PGE_2_), and TIMP-2. The pooled standardized mean difference of IL-1β and MMP-8 was −1.04 and 35.90, respectively, but the differences between periodontitis patients and healthy controls were not significant. Although the changes in salivary IL-1β and MMP-8 levels after non-surgical periodontal therapy were not significant, salivary cytokines could be used to confirm the effect of periodontal therapy or diagnose periodontal disease.

## 1. Introduction

Periodontal disease is the most common bacterial infection worldwide. It occurs mainly in adults, and a large population of people aged 30 years or older show this disease [1]. The prevalence of periodontal disease is known to increase with age in the elderly population aged 65 years or older [2]. Periodontal disease is divided into two stages. The initial stage is characterized by gingivitis, which manifests as gingival swelling and redness with bleeding. As the condition progresses to periodontitis, the second stage, inflammation, is not confined to the gingiva and spreads to the periodontal tissue, including the periodontal ligament and alveolar bone. Periodontitis is classified into mild, moderate, and severe according to the progression status [3]. Advanced periodontitis can cause tooth mobility or even tooth loss due to severe alveolar resorption [4]. Gingivitis is confined to the gingiva, which can be returned to the healthy state by appropriate oral health management, while periodontitis represents an irreversible stage that is difficult to restore to the previous healthy state since the inflammation has spread to the alveolar bone and periodontal tissue [3,5]. Thus, early diagnosis of periodontitis is very important to prevent the loss of healthy teeth due to advanced periodontitis. Furthermore, periodontitis is a risk factor for the development of osteonecrosis of the jaws in patients taking antiresorptive drugs [6]. Dental professionals can differentiate patients with advanced periodontitis based on clinical diagnostic criteria, such as probing depth, attachment loss, bleeding on probing, and radiographic findings. However, diagnosis of periodontal disease at its incipient or early stages on the basis of clinical diagnostic criteria is difficult. Accordingly, multiple trials have been conducted for early diagnosis of periodontal disease.

Saliva offers the advantage of easy and noninvasive collection and can be used as the gold standard for early detection and monitoring of periodontitis [7,8]. Saliva contains various factors, such as enzymes, growth factors, hormones, immunoglobulins, and bacteria and their products, through which oral health status can be assessed [9]. Thus, there is growing interest in biomarker studies using saliva for the selection and diagnosis of oral diseases [7]. In particular, salivary biomarkers such as inflammatory cytokines, including interleukins (ILs) and tumor necrosis factors (TNFs) [10,11,12], enzymes [13,14,15,16,17], and growth factors [18,19], have been verified for their usefulness in the diagnosis and monitoring of diseases. Many studies have attempted to identify the key salivary biomarkers for the diagnosis and selection of periodontal diseases. In particular, changes in inflammatory cytokines and enzymes have been investigated as candidate salivary biomarkers for the diagnosis of periodontal disease. Periodontitis as an inflammatory disease has been known to increase the levels of proinflammatory cytokines, including IL-1α, IL-1β, IL-6, and TNF-α [20,21,22,23,24,25,26,27]. In addition, various enzymes, such as matrix metalloproteinases (MMPs) and inflammatory mediators, are secreted by neutrophils [22,26,28,29]. The levels of salivary biomarkers, including IL-1, IL-6, and MMP-8, are reported to be significantly increased in patients with periodontitis in comparison with healthy controls [30]. Thus, this study aimed to perform a systematic review and meta-analysis for the utility of salivary biomarkers as a diagnostic tool for distinguishing patients with periodontitis and healthy controls, by examining the changes in salivary IL-1β and MMP-8 levels before and after non-surgical periodontal therapy.

## 2. Materials and Methods

The authors followed criteria established in the Preferred Reporting Items for Systematic Reviews and Meta-Analyses (PRISMA) guidelines for this review [31], and it aimed to analyze the salivary cytokine content in relation to periodontal disease and evaluate alterations in salivary biomarker levels in relation to periodontal disease after non-surgical periodontal therapy. The target search strategy was prepared on the basis of the research question; the articles to be analyzed were selected in accordance with the inclusion and exclusion criteria of the research; and the data were extracted and analyzed after quality evaluation.

### 2.1. Search Strategy

The MEDLINE and EMBASE databases were searched using relevant Medical Subject Headings (MeSH) or EMTREE terms to identify the relevant articles. The search terms confirmed by the preliminary survey were as follows: “(gingivitis OR periodontitis OR periodontal disease) AND saliva NOT caries”. Secondary searches were performed in EMBASE using the EMTREE terms “Gingivitis OR periodontitis OR periodontal disease AND saliva NOT caries” and “periodontitis” as the free text term. Using these search terms, we searched for all academic papers published until 20 August 2020, and identified those with full-text availability. The reference lists of the eventually included papers were hand-searched to identify additional relevant studies. The list of materials extracted from each site was collected in a bibliographic program; duplicate data were excluded; and the selection process was performed by applying the inclusion and exclusion criteria. Except for the cases in which the language could not be interpreted (i.e., the paper was not in English or Korean) or in cases where the full text could not be verified, all assessments were conducted by two researchers, and when the researchers’ initial assessments were different, the final decision was based on a consensus after several discussions.

### 2.2. Inclusion and Exclusion Criteria

Studies that met the following criteria were included: (a) adult participants aged 20 or older with no underlying systemic disease (population), (b) periodontal therapy or non-surgical periodontal therapy (interventions), (c) assessment of salivary cytokines related to periodontal disease was performed after treatment, and (d) a comparative study was performed between the levels of these markers before and after periodontal treatment (comparison and outcome). Exclusion criteria were as follows: studies that investigated the changes in salivary enzymes or proteins other than cytokines and MMPs and simple research studies instead of a laboratory study and secondary analysis studies.

### 2.3. Quality Assessment of the Selected Studies

The Risk of Bias in Nonrandomized Studies of Interventions (ROBINS-I) tool [32,33] was used to assess the study quality. Bias was evaluated as “yes”, “probably yes”, “probably no”, “no”, or “unclear”. The risk of bias was evaluated as “low”, “moderate”, “serious”, and “critical”. The quality evaluation of the literature was conducted by two reviewers who independently read the original text. Differences in evaluation scores were discussed to reach a final agreed-upon consensus score.

### 2.4. Population, Intervention, Comparison, and Outcome Data Extraction

Population, intervention, comparison, and outcome (PICO) data were extracted from all the included studies. Additionally, information regarding the study design and source (author and year of publication), aim of the study, and the number of groups was collected. The total number of participants, age and sex ratio were assessed as part of the participants’ characteristics. The duration of periodontal therapy and intervention details sufficient for replication were also assessed. The number of participants allocated to each group, summary data for each intervention, and control groups (as reported), including adverse events, were assessed.

### 2.5. Outcome Measures and Statistical Analysis

The effects of changes in salivary cytokine levels after non-surgical periodontal therapy were analyzed. The changes in the levels of salivary IL-1β, MMP-8, MMP-9, and TNF-α before and after treatment were compared in 11 studies. Statistical homogeneity was tested. To test the statistical homogeneity of effect sizes, a chi-squared test of homogeneity was performed, and the inconsistency index (I^2^) and Q statistics were determined. Treatment effect, measured as continuous data, was expressed as the mean difference (MD) with 95% confidence intervals for outcomes measured with the same outcome measurement instrument or as standardized mean difference (SMD) with 95% confidence intervals when different measurement instruments were used in all the studies included. Continuous outcomes, such as salivary IL-1β and MMP-8 levels, were analyzed and expressed as SMDs with 95% confidence intervals. *p* values < 0.05 for both sides were considered to be statistically significant. However, the publication bias did not suggest because the number of papers was less than 10 in the meta-analysis. Statistical analysis for the meta-analysis was performed using Comprehensive Meta-Analysis, v2.0 (Biostat, Englewood, NJ, USA).

## 3. Results

### 3.1. Study Selection

**Figure 1:**
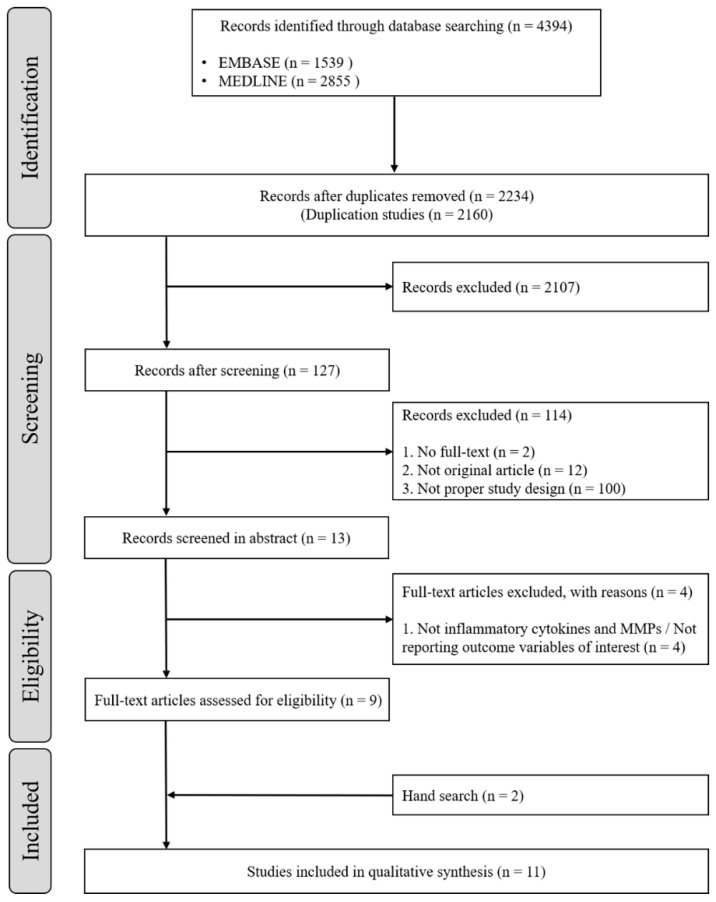
Flow diagram of the study selection process.

After full-text assessments, a total of nine studies that met the study objective were identified. By adding two manually searched papers, a total of 11 studies were finally included for this systematic review and meta-analysis (Figure 1).

### 3.2. Characteristics of the Included Studies

The characteristics of participants are demonstrated in Table 1. All participants were healthy with no symptoms of systemic diseases at the time of the research and in the past. All participants had ≥16 teeth, except third molars, and had no history of previous periodontal treatment. Pregnant and breastfeeding women were excluded. Participants were classified as having periodontal disease on the basis of evaluations of pocket depth (PD), clinical attachment loss (CAL), bleeding of probing (BOP), and alveolar bone loss in radiographs. Some studies classified periodontal disease into mild, moderate, and severe according to disease severity [20,28]. Participants with PD ≤ 3 mm, no CAL, no clinical signs of gingival inflammation, and minimal BOP scores were classified into the periodontally-healthy control group.

### 3.3. Excluded Studies

Most of the papers excluded from the final 11 were confirmed as a result of the enzyme in saliva [20] or other biomarkers other than cytokines in saliva [35,36,37,38]. The case of confirming the salivary proteome as a result [38], the case of confirming the salivary tissue inhibitor of matrix metalloproteinases (TIMPs), and MPO levels were also excluded [29].

The characteristics of studies published until 20 August 2020 that reported changes in salivary biomarkers between before and after SRP in patients with periodontal disease are summarized in Table 2. The included studies are summarized according to the participant characteristics and types of collected saliva and biomarkers. The mean age of participants in these studies varied from 34 to 61 years [24,27]. A total of 234 periodontally-healthy controls were identified after excluding two studies with no healthy control group, and the total number of patients with periodontal disease was 376. There was no significant sex-related difference in the number of participants in the studies [20,21,22,23,24,27,28]. Participants were classified as “periodontally-healthy” and those “with periodontal disease”. The included studies could be divided into those that only included patients with periodontal disease [22,27,34] and those that assessed both patients with periodontal disease and periodontally-healthy controls [20,21,23,24,25,26,28,29]. The studies that assessed periodontal disease patients alone included those that performed SRP and OHI for every participant and then compared the findings before and after the treatment [27,34]. Another study randomly divided periodontal disease patients into two groups and then compared the findings in both groups after providing SRP or OHI, respectively [22]. Shyu et al. [27] performed scaling for periodontal patients who were divided into two groups according to disease progression (non-progression and effective treatment group) and compared the findings for both groups. The studies involving both periodontal patients and periodontally-healthy controls performed SRP and OHI for periodontal patients and OHI only [20], OHI and prophylaxis [25,28,29], or no treatment for the periodontally-healthy controls [21,23,24]. Rangbulla et al. [26] provided SRP and OHI to both periodontal patients and periodontally-healthy controls. To identify the changes in biomarkers after SRP and OHI, most studies collected unstimulated saliva rather than stimulated saliva at 4 and 12 weeks after SRP and OHI. The inflammatory cytokines and enzymes used as salivary biomarkers were IL-1α, IL-1β, IL-4, IL-6, IL-8, IL-10, IL-17, IL-32, TNF-α, and TNF-β, PGE_2,_ MMP-8 and MMP-9, macrophage inflammatory protein (MIP)-1α, and TIMP-2, among which IL-1β, TNF-α, MMP-8, and MMP-9 were most commonly used to verify the changes after SRP.

### 3.4. Main Findings

The changes in salivary biomarker levels after SRP and OHI are the main findings of these 11 studies. The biomarkers showing elevated levels in the baseline saliva of patients with periodontal disease were IL-1β, IL-4, IL-6, MMP-8, and TIMP-2, while the control group showed high levels of TNF-a, IL-10, IL-17, and IL-32. The biomarkers that decreased after SRP and OHI in periodontitis patients were IL-1β, MMP-8, MMP-9, PGE_2_, and TIMP-2. After SRP and OHI, TNF-α and IL-32 levels significantly decreased while IL-1β, IL-10, and MMP-8 levels significantly increased in comparison with those in the control group. The level of IL-8 did not vary from baseline after OHI and SRP.

Among salivary biomarkers, IL-1β was associated with clinical indicators (PD, BOP, CAL, GI, and radiographic assessment). The sensitivity and specificity of predicting periodontitis on the basis of IL-1β and PGE2 levels were also reported.

Sanchez et al. [20] reported that in the mild periodontitis group, IL-1β did not increase, whereas PGE2 increased. In the moderate periodontitis group, both biomarkers significantly increased. In the severe disease group, these two biomarkers increased similar those of the moderate disease group. MMP-8 correlated with change in PD ≥ 4 mm and positive BOP.

Depending on the stage of periodontal disease progression, the biomarkers in saliva with high linkage are IL-1β in the immunologic phase and MMP-8 in the inflammatory phase [26]. These two biomarkers demonstrated a high specify and sensitivity to predict collagen and alveolar bone loss.

### 3.5. Quality Assessment

Study quality as assessed by the ROBINS-I tool is summarized in Table 3. Among the 11 included studies, nine studies were graded as low-risk and two were graded as moderate-risk. All 11 studies met the ROBINS-I criteria for case definition and showed good representativeness (Table 3).

### 3.6. Data Synthesis and Meta-Analysis

The salivary cytokines identified from each independent study assessing both periodontitis patients and healthy controls are summarized in Table 2. Although the levels of IL-1β and MMP-8 and MMP-9 were presented in seven of the 11 articles, a meta-analysis was performed using the quantitative values for IL-1β and MMP-8 presented in the studies by Rangbulla et al. [26] and Kinney et al. [28] Except for these two studies, the levels of IL-1β and MMP-8 were presented as percentages or not quantified in the other studies. Since Kinney et al. did not include quantitative values in the original article, we requested the row data from the corresponding author, which was used for meta-analysis.

**Figure 2:**
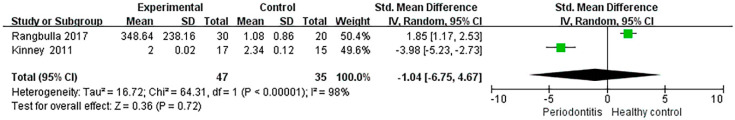
Effects on salivary IL-1β after non-periodontal surgery therapy. Note: green color, random effect interval in each study; black, total effect.

The pooled SMD of IL-1β was −1.04 [95% CI: −6.75, 4.67] with the forest plot drawn in Figure 2. For heterogeneity testing, the chi-square value was 64.31 (*p* < 0.0001), and *I*^2^ (variation in SMD attributable to heterogeneity) was 98%. Thus, the variability in the difference between periodontitis patients and healthy controls was not significant.

**Figure 3:**
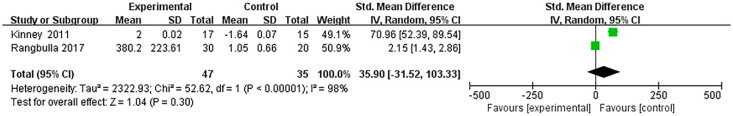
Effects on salivary MMP-8 after non-periodontal surgery therapy. Note: green color, random effect interval in each study; black, total effect.

The pooled SMD of MMP-8 was 35.90 (95% CI: −31.52, 103.33) with the forest plot drawn in Figure 3. For heterogeneity testing, the chi-square value was 52.62 (*p* < 0.0001), and *I*^2^ (variation in SMD attributable to heterogeneity) was 98%. Thus, the variability in difference between periodontitis patients and healthy controls was not significant.

## 4. Discussion

Dental professionals meet patients who are on the verge of losing a large number of teeth. The oral condition of the patients makes both the dental professionals and the patient very unfortunate. In order to prevent the condition, it is important to visit the dentist regularly for examinations and accurate diagnosis. For accurate diagnosis, periodontists conduct radiological and hand-instrument examinations. However, the clinical diagnosis by hand instrument examination is a time-consuming and difficult to both dental professionals and patients because all teeth must be assessed and recorded. Radiological examination may result in undiagnosed areas due to overlapping structures. Although these methods are generally used for clinical diagnosis of periodontal disease, it has the disadvantages of providing past disease and limited information on the progress of future periodontal disease. Therefore, a test kit for identifying bacteria and a system for testing for genetic factors related to periodontal disease has been reported. These new examination methods are difficult to alternate radiographic and hand-instrument examination, but their necessity is increasing in diagnostic examinations.

During periodontal disease, host inflammatory cells are recruited, and inflammatory cytokines, such as IL-1β, IL-6, and TNF-α, are released from fibroblasts, macrophages, connective tissue, and junctional epithelial cells. Then host-derived enzymes, MMP-8, MMP-9, and calprotectin are released by PMNs and osteoclasts, leading to connective tissue and alveolar bone degradation [28].

Various biomarkers in saliva have been proposed as candidates for the diagnosis of periodontal disease. Bacterial factors and inflammatory responses by cytokines, chemokines, and various factors that can cause periodontal disease have been identified at the saliva level. Therefor this meta-analysis systematically evaluated the salivary IL-1β and MMP-8 levels after SRP treatments in two independent studies, and the systematic review investigated the available evidence related to the differences in salivary biomarkers after non-surgical periodontal treatment. To the best of our knowledge, this is the first systematic review and meta-analysis to address these objectives. The main finding of the systematic review of 11 studies was that the inflammatory cytokines and enzymes used as salivary biomarkers were IL-1α, IL-1β, IL-4, IL-6, IL-8, IL-10, IL-17, and IL-32, TNF-α, TNF-β, PGE_2,_ MMP-8, MMP-9, MIP-1α, and TIMP-2. IL-1β, TNF-α, MMP-8, and MMP-9 were the most commonly used salivary biomarkers used to identify the changes after SRP. The results of the meta-analysis confirmed that salivary IL-1β and MMP-8 levels were not significantly different before and after SRP treatment.

A previous study reported that among the salivary biomarkers, IL-1β, MMP-8, MMP-9, and OPG demonstrated the highest correlation with disease status [39]. Further, the use of multiple time-points with two-month intervals for assessment of saliva biomarkers allows for an improved understanding of biomarker fluctuations over time. In this review, most studies collected clinical data and saliva samples once at one or three months after SRP, and several studies obtained measurements twice at one and three months. Various regenerative surgical modalities have been suggested and examined for the regeneration of periodontal specific tissues: alveolar bone, cementum, periodontal ligament, and gingiva. These studies assessed the recovery of the alveolar bone or periodontal tissue after four or eight weeks to verify the degree of healing [40].

Using a meta-analytical approach, a previous study found that MMP-8, MMP-9, IL-1β, IL-6, and Hb were salivary biomarkers with good capability to detect periodontitis in systemically healthy subjects. MMP-8 and IL-1 β are the most researched biomarkers in related researches, both showing clinically fair effectiveness for the diagnosis of periodontitis [41]. In this study, these two markers were identified most frequently among 11 studies.

IL-1β is a prototype “multifunctional” proinflammatory cytokine that plays a major role in acute and chronic inflammation [42]. Fine et al. longitudinally evaluated periodontal disease progression in children at risk for aggressive periodontitis and reported that IL-1β demonstrated a high specificity and sensitivity to predict alveolar bone loss [43]. IL-1β stimulated endothelial cells to induce selectins, which facilitate recruitment of leukocytes, activate macrophage IL-1 production, stimulate production of inflammatory mediators (e.g., PGE_2_), cause MMP expression, enhance osteoclast formation and activity, stimulate the apoptosis of matrix-producing cells leading to inflammation, connective tissue breakdown, bone loss, and limited repair of periodontium [44].

MMPs are proteolytic enzymes belonging to the zinc protease superfamily and are involved in physiological degradation of extracellular matrix proteins and basement membranes. They can be categorized into several groups [45]. Salivary biomarkers, specifically MMP-8, MMP-9, OPG, and IL-1β, are present in low concentrations and can predict stability in 78% of individuals who are clinically stable during disease monitoring.

The pooled SMDs of salivary IL-1β and MMP-8 were not significantly confirmed after SRP treatments. Although salivary changes were checked 4–16 weeks after SRP, no significant difference was found. This result may be attributable to the minimal changes in the salivary levels of IL-1β and MMP-8 presented in the study by Kinney et al. [28], which was included in this meta-analysis. A meta-analysis performed by Zhang et al. [46] reported that salivary MMP-8 levels were significantly higher in periodontitis patients than in healthy controls overall. On the other hand, no significant changes were observed in serum biomarker levels after non-surgical periodontal treatment in pregnant women with periodontitis [47]. Furthermore, analyses of serum biomarkers have been reported to yield inconsistent findings across individuals and were largely not sustainable [48]. A previous systematic review and meta-analysis indicated that MIP-1*α* had excellent diagnostic accuracy while IL-1β and IL-6 had acceptable diagnostic values. However, one study only evaluated the biomarkers considered to be excellent, which may reduce the robustness of the results [49]. As shown in this study, further studies are needed to determine the salivary biomarkers that can be used for the diagnosis of periodontal disease and confirmation of treatment effects. MMP-8 showed a significant difference between the treatment and control groups. However, there is a lack of studies investigating the changes in salivary biomarkers after SRP. Thus, a meta-analysis including more studies will be needed to identify significant differences.

IL-8 has a role as a major mediator of the inflammatory response and chemoattractant for neutrophils. However, in the studies included in this review, no significant differences were found before and after treatment [22,50]. Yang et al. [21] showed that IL-17 level was higher in chronic periodontitis group and was correlated with variation in the microbial parameters. IL-17 is a pro-inflammatory cytokine produced by T-helper 17 cells, which recruit neutrophils and macrophages to participate in and amplify inflammatory reaction [51].

The levels TNF-α and IL-32 in gingival crevicular fluid and saliva were higher in chronic periodontitis patients, and then after treatments, these two biomarkers decreased. However, the levels of IL-10 were lower in patients and the IL-10 levels were higher after treatments [23]. Previous studies demonstrated that TNF-α is one of the most influential cytokines and plays a key part in pathogenesis of several serious and persistent inflammatory ailments [52]. Moreover, IL-10 is the powerful anti-inflammatory cytokine and might also play an important role in the control and development of periodontal inflammation [53]. IL-32 is evaluated for one of the newest discoveries cytokine and has proinflammatory properties and is stimulated by activated T-lymphocytes and activated natural killer cells. In addition, IL-32 stimulates the production of osteoclasts, without the need for autonomous RANKL generation [54].

Meschiari et al. [29] reported that using zymography gelationlytic activity of MMP-9 forms may be related to lower local inflammation, represented by improvement of the clinical parameters. The reduction in biofilm formation by scaling leads to lower levels of pro-inflammatory cytokines [55]. The reduction in tissue degradation after PD treatment seems to be associated with decreased MMP-9 activity. However, there is no significant difference in MMP-9 activity before and after treatment [29].

In conclusion, no changes were observed in salivary IL-1β and MMP-8 levels after SRP in this meta-analysis. However, in the 11 studies included in this study, IL-1β, MMP-8 and TNF-α in saliva were the most frequently observed biomarkers in periodontal disease patients compared to healthy controls. In addition, many studies have suggested the possibility of a point-of-care device using biomarkers in saliva. However, further interpretations based on these results should be performed with caution. The limitations of this study are as follows: each study included a small number of participants; not many studies investigated the changes in salivary biomarkers after SRP; the criteria for the diagnosis of periodontal disease were not identical among the included studies; and depending on the classification used, patient groups were divided into severe or mild periodontitis.

In summary, this systematic review shows that while salivary cytokine levels related to periodontal disease after treatment did show quantitative differences, the meta-analysis did not show significant differences. Thus, collection of saliva biomarkers could offer potential for the prediction of periodontal disease progression in large patient populations.

## 5. Conclusions

There were insufficient data to conduct a meta-analysis on the effect of non-surgical periodontal therapy on changes in salivary IL-1β and MMP-8 levels. The meta-analysis showed no statistically significant decrease or alterations in the levels of IL-1β and MMP-8 after non-surgical periodontal therapy between the treatment and healthy control groups. However, we confirmed that the inflammatory cytokines and enzymes used as salivary biomarkers were IL-1α, IL-1β, IL-4, IL-6, IL-8, IL-10, IL-17, IL-32, TNF-α, TNF-β, PGE2, MMP-8, MMP-9, MIP-1α, and TIMP-2, and that IL-1β, TNF-α, MMP-8, and MMP-9 were most commonly used salivary biomarkers to identify the changes after SRP, indicating the potential applicability of saliva for the diagnosis of periodontal disease.

## Figures and Tables

**Table 1:** Participant characteristics in the included articles.

Author (Year)	Participant Characteristics	
	Patient Group	Control Group
Yoshie et al. (2007) [34]	38 non-smokers and 11 smokers. The mean number of teeth present in all patients was 26.4. None of the participants had a history or current signs of systemic disease.	N/A
Sexton et al. (2011) [22]	≥18 erupted teeth. None of the participants had a history or current signs of systemic disease and had not received periodontal treatment or antibiotic therapy previously. No pregnant or lactating patients were included in the study.	
	<Criteria for periodontal disease>	
	PD ≥ 5 mm, CAL of ≥3 mm, and BOP score of ≥2.	
Kinney et al. (2011) [28]	≥20 erupted teeth. None of the participants had a history or current signs of systemic disease and had not received periodontal treatment or antibiotic therapy previously. No pregnant or lactating patients were included in the study.	
	<Criteria for periodontitis>	<Criteria for periodontal health and gingivitis>
	Mild: ≥4 sites with evidence of radiographic bone loss, ≤30% sites with CAL > 3 mm, and ≥4 sites with PD > 4 mm. Moderate-severe: ≥4 sites with evidence of radiographic bone loss, >30% of sites with CAL > 3 mm, and ≥4 sites with PD > 4 mm.	Periodontally-healthy: <3 mm of CAL, no PD of >4 mm, no radiographic alveolar bone loss, and BOP ≤ 20%. Gingivitis: <3 mm of CAL, no PD of >4 mm, no radiographic alveolar bone loss, and BOP > 20%.
Kaushik et al. (2011) [24]	Non-smoking. None of the participants had a history or current signs of systemic disease and had not received periodontal treatment or antibiotic therapy previously.	
	<Criteria for periodontitis>	<Criteria for periodontal health>
	Moderate-severe: ≤2 teeth missing in each quadrant, ≥30% sites with PD ≥ 4 mm; ≥20% sites with CAL > 2 mm; ≥30% sites showing BOP; and visible radiographic evidence of bone loss.	<10% of sites with BOP, no sites with PD ≥ 4 mm, no CAL > 2 mm, and no visible radiographic evidence of bone loss.
Sánchez et al. (2013) [20]	Non-smoking; no pregnancy. None of the participants had a history or current signs of systemic disease and had not received periodontal treatment or antibiotic therapy previously.	
	<Criteria for periodontitis>	<Criteria for periodontal health>
	Mild: <2 sites with CAL ≥ 4 mm and <2 sites with PD ≥ 5. Moderate: ≥2 sites with CAL ≥ 4 mm and ≥2 sites with PD ≥ 5. Severe: ≥2 sites with CAL ≥ 6 mm and ≥2 sites with PD ≥ 5.	Absence of periodontal disease.
Meschiari et al. (2013) [29]	≥20 erupted teeth. Non-smoking (current or former smoker for <10 years); no pregnancy. None of the participants had a history or current signs of systemic disease and had not received periodontal treatment or antibiotic therapy previously.	
	<Criteria for periodontal disease>	<Criteria for periodontal health>
	At least 2 teeth with PD ≥ 5 mm, CAL ≥ 6 mm, and visible radiographic evidence of bone loss.	Absence of periodontal disease.
Prakasam et al. (2014) [25]	Non-smoking. None of the participants had a history or current signs of systemic disease and had not received periodontal treatment or antibiotic therapy previously.	
	<Criteria for chronic periodontitis>	<Criteria for periodontal health>.
	Moderate to severe: ≥30% sites having ≥4 mm CAL, severe clinical inflammation, and high BOP scores.	no CAL, no overt clinical signs of gingival inflammation, and minimal BOP scores.
Shyu et al. (2015) [27]	≥16 functional teeth. Systemically healthy status.	N/A
	<Criteria for chronic periodontitis>	
	≥6 pockets with PD of >5 mm. Two subgroups after treatment (scaling). Nonprogress treatment group: differences in the percentage of patients showing >7 mm PD increase between the initial clinical treatment and after the completion of 4 weeks of clinical treatment. Effective treatment group: remaining patients.	
Yang et al. (2016) [21]	≥20 natural teeth (including at least 4 molar teeth). Non-smoking; no pregnancy. None of the participants had a history or current signs of systemic disease and had not received periodontal treatment or antibiotic therapy previously.	
	<Criteria for chronic periodontitis>	<Criteria for periodontal health>
	PD of ≥4 mm; CAL of ≥2 mm; alveolar bone destruction of >30%.	PD of ≤3 mm, CAL of ≤1 mm, and no sulcular bleeding index.
Öngöz et al. (2017) [23]	≥20 natural teeth. Non-smoking; no pregnant and lactating patients were included. None of the participants had a history or current signs of systemic disease and had not received dental (periodontal and orthodontic) treatment or antibiotic therapy previously.	
	<Criteria for chronic periodontitis>	<Criteria for periodontal health>
	GI rating of >1, ≥6 teeth with PD of ≥5 mm, and BOP in at least two separate regions.	PD ≤ 3 mm, a GI rating of zero, no indications of CAL, no visible radiographic evidence of bone loss.
Rangbulla et al. (2017) [26]	<periodontitis criteria>	<Criteria for periodontal health>
	≥20 teeth. Non-smoking. None of the participants had received professional oral prophylaxis during the past 12 months or antibiotic therapy. PD ≥ 5 mm, CAL ≥ 4 mm.	None of the participants had a history or current signs of systemic disease. No pregnant and lactating were included in this study.

BOP, bleeding on probing; CAL, clinical attachment loss; PD, pocket depth; GI, gingival index.

**Table 2:** Characteristics of the included articles.

Author (year)	Patient Group				Control Group				Sample Collection	Inflammatory Cytokines	Main Findings
	Age (years; Mean ± SD)	N (Male/Female)	Treatment	Period (Saliva Sampling after Treatment)	Age (years; Mean ± SD)	N (Male/Female)	Treatment	Period (Saliva Sampling after Treatment)			
Yoshie et al. (2007) [34]	CP: 55.1 ± 2.0	49 (24/25)	SRP	At baseline and 4 weeks	N/A	N/A	N/A	N/A	SWS	IL-1	IL-1A allele 2 non-carriers displayed a significant decrease in salivary AST and ALT levels; the carriers did not show any changes in the salivary levels of the enzymes after scaling.
Sexton et al. (2011) [22]	CP: 40.3 ± 10.0	35 (26/9)	SRP and OHI	At 0, 16, and 28 weeks	CP: 47.3 ± 8.8	33 (21/12)	OHI	At week 0, 16 and 28	UWS	IL-1β, IL-8, MMP-8, MIP-1α, and TNF-α	Baseline TNF-α levels changed significantly at both follow-up visits (16 and 28 weeks), regardless of the treatment group. IL-1β and MMP-8 levels decreased significantly from baseline (*p* < 0.04) in the SRP group only. MMP-8 and MIP-1α levels were significantly reduced in comparison with those in the non-responders to treatment (*p* = 0.01, 0.05 respectively). In receiver-operating characteristic analyses, MMP-8 produced the highest area under the curve (≥0.7; *p* = 0.01).
Kinney et al. (2011) [28]	Mild CP: 54; Moderate to severe CP: 50	Mild: 24 (11/13); Modertate to Severe: 20 (7/13)	SRP and OHI	Bi-monthly over a 12-month period	Periodontally-healthy: 46 Gingivitis: 46	Periodontally-healthy: 15(9/6) Gingivitis: 24(10/14)	Prophylaxis and OHI	Bi-monthly over a 12-month period	UWS	IL-1β, MMP-8, and MMP-9	Moderate to severe periodontitis patients demonstrated reduction of MMP-8, MMP-9, and IL-1β at 12 months in comparison with baseline (*p* < 0.05).
Kaushik et al. (2011) [24]	Moderate-to-severe CP: 34.9 ± 6.4	28 (8/20)	SRP and OHI	Before and 1 month	Periodontally-healthy: 33.6 ± 4.1	24 (9/15)	No treatment	Before and 1 month	UWS	IL-1β	IL-1β levels in periodontitis patients reduced significantly post-treatment but were still significantly higher than the baseline values of controls. IL-1β showed a significant positive correlation with percentage probing depth, bleeding on probing, gingival index, and periodontal index.
Sánchez et al. (2013) [20]	Mild periodontitis: 38.3 Moderate periodontitis: 41.6 Severe periodontitis: 46.8	Mild 18 (14/4) Moderate 21 (17/4) Severe 20 (14/6)	SRP and OHI	At 3 months	Periodontally-healthy: 34.3	15 (10/5)	OHI	At 3 months	UWS	IL-1β and PGE_2_	IL-1β and PGE_2_ levels reduced significantly post-treatment. With a selected threshold of 212 pg/mL, salivary IL1-β predicted periodontitis with 78% sensitivity and 100% specificity. With a selected threshold of 121 pg/mL, salivary PGE_2_ predicted periodontitis with 78% sensitivity and 91% specificity.
Meschiari et al. (2013) [29]	None	19 (none)	SRP	Before and after 3 months of treatment	Periodontally-healthy: none	11 (none)	OHI and prophylaxis	Before and after 3 months of treatment	SWS	MMP-8 and TIMP-2	MMP-8 and TIMP-2 baseline concentrations in the periodontal group were significantly higher than those in the controls, but their concentrations decreased after non-surgical therapy.
Prakasam et al. (2014) [25]	Moderate to severe CP: 40.80 ± 10.07	18 (9/9)	SRP	At 1 and 6 weeks	Periodontally-healthy: 28.00 ± 2.94	18 (10/8)	Prophylaxis	Approximately 1–2 weeks	UWS	IL-4, IL-6, IL-10, and IL-17	IL-4 and IL-6 levels were significantly higher and IL-10 and IL-17 levels were significantly lower in chronic periodontitis patients in comparison with healthy controls. IL-4 levels were lower at 6 weeks post-SRP. IL-6 and -17 levels did not change post-SRP. IL-10 levels were significantly higher at 6 weeks post-SRP.
Shyu et al. (2015) [27]	CP Nonprogress (NP) treatment group: 61.5 Effective treatment (ET) group: 56.0	NP group 12 (5/7) ET group 10 (4/6)	Scaling	Before and after scaling	N/A	N/A	N/A	N/A	UWS	IL-1α, 1β, 6, 8, TNF-α, and β	Baseline IL-1α and scaling-stimulated IL-1α showed a positive correlation (r = 0.66 and *p* < 0.01). Baseline IL-1β and scaling-stimulated IL-1β also showed a positive correlation (r = 0.44 and *p* = 0.04). Scaling-stimulated IL-6 was significantly correlated with baseline IL-1α, IL-1β, IL-6, and TNF-α. The differences in IL-1α, IL-6, and IL-8 were significantly higher in the ET group than in the NP group.
Yang et al. (2016) [21]	CP: 36.593 ± 11.502	45 (19/26)	SRP and OHI	At baseline and at 1 and 3 months	Periodontally-healthy: 35.827 ± 8.012	47 (19/28)	No treatment	N/A	UWS	IL-17	IL-17 levels significantly reduced post-treatment in comparison with the baseline (before treatment) levels, especially at 3 months than at 1 month after treatment.
Öngöz et al. (2017) [23]	Mild to moderate CP: 39.44 ± 3.15	27 (14/13)	SRP	Before and at 4 weeks	Periodontally-healthy: 37.30 ± 3.80	27 (12/15)	No treatment	Before assessment	UWS	IL-10, IL-32, and TNF-α	TNF-a and IL-32 levels in the periodontitis group were significantly lower after treatment compared with the baseline levels, but IL-10 levels were significantly higher.
Rangbulla et al. (2017) [26]	Moderate to severe CP: none	30 (none)	SRP and OHI	Before and 12 weeks	Periodontally-healthy: none	20 (none)	SRP and OHI	Before and 12 weeks	UWS	IL-1β and MMP-8	IL-1β and MMP-8 levels in periodontitis patients reduced significantly after oral prophylaxis, but were still significantly higher than the baseline values of controls.

AST: aspartate aminotransferase; ALT: alanine aminotransferase; CP: chronic periodontitis, SRP: scaling and root planning, OHI: oral hygiene instruction, IL: interleukin, MMP: matrix metalloproteinase, MIP: macrophage inflammatory protein, TNF: tumor necrosis factor, PGE_2_: prostaglandin E2, TIMP: tissue inhibitor of matrix metalloproteinases, UWS: unstimulated whole saliva, SWS: stimulated whole saliva.

**Table 3:** Quality assessment of the included studies (ROBINS-I tool).

Authors (Year)	Confounding Bias	Selection Bias	Classification Bias	Intervention Bias	Missing Data Bias	Measurement Bias	Reporting Bias	Overall Bias
Yoshie et al. (2007) [34]	PN	N	N	N	N	N	N	Low
Sexton et al. (2011) [22]	N	N	N	PN	UN	N	N	Low
Kinney et al. (2011) [28]	N	N	N	PN	N	N	N	Low
Kaushik et al. (2011) [24]	N	N	N	PN	N	N	N	Low
Sánchez et al. (2013) [20]	UN	N	N	PY	UN	N	N	Moderate
Meschiari et al. (2013) [29]	UN	N	N	PN	PN	N	N	Moderate
Prakasam et al. (2014) [25]	PN	N	N	N	N	N	N	Low
Shyu et al. (2015) [27]	N	N	N	N	N	N	N	Low
Yang et al. (2016) [21]	N	N	N	N	N	N	PY	Low
Öngöz et al. (2017) [23]	N	N	N	PN	N	N	N	Low
Rangbulla et al. (2017) [26]	PN	N	N	N	UN	N	N	Low

Each domain is evaluated with one of the following: PY = “probably yes”, PN = “probably no”, N = “no”, and UN = “unclear”. The categories of overall bias for each study are low, moderate, serious, and critical risk of bias.

## Abbreviations

IL: interleukin; ILs: interleukins; MMP: matrix metalloproteinase; MMPs: matrix metalloproteinases; TNFs: tumor necrosis factors; SRP: scaling and root planning; OHI: oral hygiene instruction; PGE_2_: prostaglandin E_2_; PRISMA: Preferred Reporting Items for Systematic Reviews and Meta-Analyses; MeSH: Medical Subject Heading; ROBINS-I: Risk of Bias in Nonrandomized Studies of Interventions; PICO: Population, intervention, comparison, and outcome; MD: mean difference; SMD: standardized mean difference; PD: pocket depth; CAL: clinical attachment loss; BOP: bleeding of probing; GI: gingival index; AST: aspartate aminotransferase; ALT: alanine aminotransferase; CP: chronic periodontitis; MIP: macrophage inflammatory protein; TIMP: tissue inhibitor of matrix metalloproteinase;, UWS: unstimulated whole saliva; SWS: stimulated whole saliva.
